# Computed tomography angiography‐guided analysis of morphologic properties of the thoracic aorta and arch branches among the adult population: A cross‐sectional study

**DOI:** 10.1002/hsr2.70017

**Published:** 2024-08-29

**Authors:** Ahmad Hosseinzadeh, Ali Tajaddini, Seyed Hamed Jafari, Zahra Mohammadi, Farzad Dalfardi, Hossein Fatemian, Reza Shahriarirad

**Affiliations:** ^1^ Thoracic and Vascular Surgery Research Center Shiraz University of Medical Science Shiraz Iran; ^2^ Department of Surgery Shiraz University of Medical Sciences Shiraz Iran; ^3^ Medical imaging research center Shiraz University of medical sciences Shiraz Iran; ^4^ School of Medicine Shiraz University of Medical Sciences Shiraz Iran

**Keywords:** Aortic Arch, Bovine Arch, Brachiocephalic Trunk, Common Carotid Artery, Computed tomographic angiography, Thoracic Aorta

## Abstract

**Background and Aims:**

Before performing any surgical or endovascular procedure, the anatomical classification of the patient is essential for treatment planning. Computed Tomographic Angiography (CTA) is a standard method to evaluate thoracic aortic anatomy and associated variations.

**Methods:**

This cross‐sectional, descriptive study was performed among adult participants without any peripheral vascular diseases undergoing thoracic CTA. Demographic data were collected along with factors retrieved from the patients CTA, such as the type of aortic arch, length, and diameter of ascending and descending aorta, the diameter of the main branches and the distance between branches, the angles by which the main arteries rise from their origins, and left anterior oblique angle of the aortic arch.

**Results:**

164 patients, with a mean age of 57 ± 19.3 years, entered the study. 53 (33.8%) had a bovine arch, which was mostly observed among males. A decrease in the frequency of type I arch and also an increase in the frequency of type 2 and 3 arches was observed with the increase in age (*p* < 0.001). The BCT diameter had a direct association with both left common carotid artery diameter (*r* = 0.478, *p* < 0.001) and left subclavian artery diameter (*r* = 0.470, *p* < 0.001). The length of the descending aorta had a direct correlation (*r* = 0.294, *p* < 0.001) with the length of the ascending aorta.

**Conclusion:**

Complex endovascular interventions are vital method in treating aorta, head, and neck pathologies. Accurate knowledge of thoracic aortic anatomy is becoming crucial for diagnosis and intervention planning.

## 1 INTRODUCTION

With the growing aging population worldwide and a favorable public trend for a sedentary lifestyle, the prevalence of Cardiovascular Diseases (CDs) has been escalating significantly,^1^ to a level that CDs are considered to be the leading cause of death worldwide.^2^ Current issues have led to advances in medical, surgical, and endovascular technologies through less invasive procedures associated with a lower morbidity and mortality rate.^3^ Management and treatment of CDs have been radically facilitated by the breakthroughs of these procedures compared to traditional methods of open surgeries.^4^, ^5^, ^6^, ^7^ However, such minimally invasive procedures can occasionally be challenging due to the aorta's unique anatomy, which might be the most important reason for practical failure.^8^, ^9^, ^10^ Hence, the morphology of the aorta has a key role in performing endovascular procedures such as Thoracic Endovascular Aortic Repair (TEVAR), and it's highly essential to have a meticulous conception of its anatomy.

While performing TEVAR, it might be challenging to achieve adequate stent‐graft apposition regarding the aortic wall and synchronously maintain blood flow due to the proximity of the supra‐aortic branches.^8^ This may lead to alteration in dynamic blood flow and, in the worst‐case scenario, more fatal complications such as emboli, thrombosis, endo‐leaks, and even aortic wall damage.^11^, ^12^, ^13^

Usually, three vessels arise from the Aortic Arch (AA), including the Brachiocephalic Trunk (BCT) (which further bifurcates into the Right Subclavian Artery (RSA) and the Right Common Carotid Arteries), the Left Common Carotid Artery (LCCA), and the Left Subclavian Artery (LSA). Some other notable variations in this aspect are as follows: LCCA might originate from the BCT, the BCT might not form; thus, all four main vessels might arise from the AA, and the left vertebral artery might originate directly from the arch of the aorta, etc.^14^ Apart from different anatomic variations of AA and its branches,^15^, ^16^, ^17^ these structures differ in terms of diameter, the distance of the origins, and the location which they arise.^18^, ^19^

A study by Jakanani et al. in 2010 on investigating the frequency of different aortic anatomy variations revealed that 643 out of 861 (74%) Chest computed tomography (CT) scans and chest aortic examinations had a conventional configuration. Meanwhile, the most common anatomical type was the same origin of the brachiocephalic and LCCA (Bovine Arch) that occurred in 20% of participants.^20^ Moreover, a review of 20,030 cases done by Recto et al. reported that 80% had normal anatomy, and the bovine arch as the most frequent variation accounted for 10.1%.^21^

Before performing any surgical or endovascular procedure, the anatomical classification of the patient is essential for the planning of treatment. In other words, to achieve a detailed vision of each patient's anatomy, one must have acquaintance with normal anatomy and different variations of the target organ. Therefore, conducting radiologic, including CT Angiography (CTA) studies, is a valuable way to evaluate the vascular anatomy and variations among the population.^22^ In this study, we aimed to assess the anatomy of the aorta through CTA in adult patients referred to the vascular surgery department in a referral center in Shiraz, Iran, to provide elementary evidence for future development of endovascular procedures, especially in the AA.

## 2 METHODS AND MATERIALS

This study followed a cross‐sectional and descriptive protocol and was performed on adult patients who were referred to the Vascular Surgery Department of our University affiliated hospital and underwent thoracic CTA for various reasons and without any peripheral vascular diseases. The study protocol was explained to each patient, and informed consent was obtained before entering the study.

Patient's demographic data, including age, gender, and comorbid disease, were extracted from their records. A 16‐slice CT scan was utilized in this study, and subsequently, the images were analyzed by two experts in the field of vascular surgery; in case of any disparity between the numbers obtained from the images, a single expert radiologist assessed the images. Various factors such as the type of AA,^23^ length and diameter of ascending and descending aorta, the diameter of the main branches and the distance between branches, the angles by which the main arteries rise from their origins, and left anterior oblique angle of AA were assessed in the images. We utilized the sagittal view for assessing this distance due to its optimal perspective. This particular distance is essential to be approximated since the application of the traditional TEVAR requires a proximal and distal landing zone of at least 2 cm.^24^

Obtained data were entered into SPSS version 26, and the mean and standard deviation (SD) of the quantitative variables as well as the frequency and percentages (%) of the qualitative variables were reported. The Chi‐square test or Fisher's exact test was used to compare categorical data, while the Person correlation and independent‐sample t‐test were used to evaluate continuous variables. A P‐value of less than 0.05 was considered statistically significant.

## 3 RESULTS

A total of 164 patients entered the study, with a mean age of 57 years (SD: 19.3, range 20–94). The morphometric parameters measured in the patients are summarized in Table 1.

**Table 1:** Clinical and morphometric parameters measured in the patients.

Variable		Value; *N* = *164*
Age (years)		57.0 ± 19.3 (20–94)
Sex	Male	111 (67.7)
	Female	53 (32.3)
Comorbid disease	Hypertension	47 (28.7)
	Diabetes	28 (17.1)
Type of Aortic Arch	One	63 (42.6)
	Two	67 (45.3)
	Three	18 (12.2)
Proximal part of ascending aorta		32.1 ± 25.3 (20.7–331.0)
Proximal part of descending aorta		24.6 ± 4.0 (8–38.8)
Distal part of ascending aorta		31.4 ± 4.6 (21.9–48.0)
Distal part of descending aorta		22.6 ± 3.9 (16.0–38.0)
Brachiocephalic diameter		13.4 ± 2.2 (7.4–19)
Left subclavian diameter		10.9 ± 2.5 (4.1–19.1)
Angle of the arch of aorta		66.1 ± 7.0 (43.0–87.0)
Ascending aorta length		63.0 ± 13.0 (4.2–108.0)
Descending aorta length		209.0 ± 29.2 (138–298.3)
Left carotid diameter		8.5 ± 1.5 (5.0–12.8)
Brachiocephalic‐left carotid distance		2.2 ± 2.6 (1.0–12.4)
Left carotid‐left subclavian distance		8.6 ± 4.5 (1.7–24.0)
Incidental finding		32 (19.9)
Bovine Arch		53 (33.8)

*Note*: Values are reported as mean ± Standard deviation (range) or frequency (%).

Moreover, 157 patients were studied regarding the existence of bovine arch, of whom 53 (33.8%) had bovine arch (type II), meaning that the BCT and LCCA originated from the same spot, and 104 patients (63.4%) did not have this condition. The bovine arch was mostly observed among male patients. The association between the factors and bovine arch are represented in Table 2.

**Table 2:** Association between the clinical and morphometric parameters measured in the patients and Bovine Arch.

Variable		Bovine Arch		*P*‐value
		Yes; *n* = *53*	No; *n* = *104*	
Age (years) mean Std. deviation		59.7 ± 17.5	55.8 ± 19.7	0.228
Sex	Male	37 (34.9)	69 (65.1)	0.721
	Female	16 (31.4)	35 (68.6)	
Comorbid disease	Hypertension	10 (22.7)	34 (77.3)	0.090
	Diabetes	6 (21.4)	22 (78.6)	0.185
Type of Aortic Arch	One	29 (46.0)	34 (54.0)	0.056
	Two	19 (28.8)	47 (71.2)	
	Three	4 (22.2)	14 (14.7)	
Proximal part of ascending aorta		29.8 ± 4.3	33.3 ± 31.4	0.435
Proximal part of descending aorta		23.9 ± 3.6	24.9 ± 4.6	0.172
Distal part of ascending aorta		31.1 ± 4.7	31.5 ± 4.5	0.601
Distal part of descending aorta		22.3 ± 3.3	22.8 ± 4.1	0.484
Brachiocephalic diameter		13.0 ± 2.4	13.5 ± 2.1	0.157
Left subclavian diameter		10.5 ± 2.2	11.2 ± 2.5	0.074
Angle of the arch of aorta		66.1 ± 7.1	66.1 ± 7.0	0.991
Ascending aorta length		63.8 ± 11.7	63.0 ± 12.3	0.689
Descending aorta length		210.2 ± 24.7	210.7 ± 22.8	0.909
Left carotid diameter		7.9 ± 1.4	8.7 ± 1.6	**0.002**
Brachiocephalic‐left carotid distance		0.0	3.5 ± 2.4	**<0.001**
Left carotid‐left subclavian distance		9.8 ± 5.1	8.0 ± 4.1	**0.020**
Incidental finding		4 (14.3)	24 (85.7)	**0.016**

*Note*: Values are reported as mean ± Standard deviation (range) or frequency (%).

Bold values indicate significant association.

The Pearson correlation test was performed for pairwise evaluation of the variables and the results (Table 3). The BCT diameter had a direct association with both LCCA diameter (*r* = 0.478, *p* < 0.001) and LSA diameter (*r* = 0.470, *p* < 0.001). Moreover, the length of descending aorta had a direct correlation (*r* = 0.294, *p* < 0.001) with the length of ascending aorta.

**Table 3:** Pearson correlation between morphometric parameters measured in the patients (AA: Ascending aorta; DTA: descending thoracic aorta; BCA: brachiocephalic artery; LCC: left common carotid artery; LSA: left subclavian artery).

**Proximal of AA**	0.04; 0.60											
**Proximal of DTA**	**0.39;** <**0.001**	0.01; 0.10										
**Distal part of AA**	**0.41;** <**0.001**	0.09; 0.30	**0.56;** <**0.001**									
**Distal part of DTA**	**0.402;** <**0.001**	0.03; <0.75	**0.65;** <**0.001**	**0.49;** <**0.001**								
**BCA diameter**	0.06; 0.44	0.09; 0.31	**0.36;** <**0.001**	**0.41;** <**0.001**	**0.29; 0.001**							
**LCC diameter**	−0.05; 0.51	−0.01; 0.90	0.15; 0.08	**0.25;0.002**	0.13; 0.12	**0.48;** <**0.001**						
**LSA diameter**	0.09; 0.26	−0.01; 0.93	**0.31;** <**0.001**	**0.37;** <**0.001**	**0.33;** <**0.001**	**0.47;** <**0.001**	**0.47;** <**0.001**					
**Aorta Arch Angle**	**0.16; 0.05**	−0.001; 0.99	0.05; 0.59	0.07; 0.38	0.22; 0.10	−0.04; 0.66	−0.04; 0.63	−0.12; 0.15				
**AA length**	**0.30;** <**0.001**	0.09; 0.29	**0.37;** <**0.001**	**0.52;** <**0.001**	**0.44;** <**0.001**	**0.36;** <**0.001**	**0.17; 0.045**	**0.27; 0.001**	0.09; 0.30			
**DTA length**	**0.28; 0.001**	0.08; 0.33	**0.37;** <**0.001**	**0.40;** <**0.001**	**0.50;** <**0.001**	**0.21; 0.01**	0.13; 0.12	**0.18; 0.03**	0.02; 0.78	**0.29;** <**0.001**		
**BCA‐LCC distance**	−0.02; 0.84	−0.003; 0.97	0.16; 0.054	**0.17; 0.047**	**0.36;** <**0.001**	0.15; 0.08	**0.26; 0.002**	**0.21; 0.01**	−0.02; 0.81	0.16; 0.053	0.07; 0.43	
**LCC‐LSA distance**	−0.02; 0.86	0.08; 0.35	−0.06; 0.46	−0.07; 0.39	−0.05; 0.52	−0.15; 0.08	−0.02; 0.79	0.06; 0.48	−0.05; 0.55	−0.04; 0.61	−0.11; 0.18	−0.05; 0.59
**Pearson Correlation; P‐value**	**Age**	**Proximal of AA**	**Proximal of DTA**	**Distal part of AA**	**Distal part of DTA**	**BCA diameter**	**LCC diameter**	**LSA diameter**	**Aorta Arch Angle**	**AA length**	**DTA length**	**BCA‐LCC distance**

The morphological features of the patient's association with their comorbid disease are demonstrated in Tables 4 and 5.

**Table 4:** Evaluation of morphometric parameters measured in the patients based on hypertension.

Variable		Hypertension		*P*‐value
		Yes; *n* = 44	No; *n* = 113	
Age (years)		60.0 ± 19.6	55.8 ± 19.1	0.22
Sex	Male	29 (26.1)	89 (73.9)	0.36
	Female	18 (34.0)	35 (66.0)	
Type of Aortic Arch	One	13 (20.6)	50 (79.4)	0.29
	Two	18 (26.9)	49 (73.1)	
	Three	7 (38.9)	11 (61.1)	
Bovine Arch	Yes	10 (18.9)	43 (81.1)	0.09
	No	34 (32.7)	70 (67.3)	
Proximal part of ascending aorta		31.2 ± 4.4	32.4 ± 29.1	0.80
Proximal part of descending aorta		24.6 ± 5.5	24.6 ± 3.8	0.90
Distal part of ascending aorta		32.5 ± 4.5	31.1 ± 4.6	0.09
Distal part of descending aorta		23.7 ± 4.1	22.2 ± 3.8	**0.048**
Brachiocephalic diameter		14.0 ± 2.3	13.1 ± 2.2	**0.02**
Left subclavian diameter		11.4 ± 2.6	10.8 ± 2.3	0.15
Angle of the arch of aorta		64.5 ± 7.9	66.7 ± 6.6	0.10
Ascending aorta length		65.0 ± 13.6	62.3 ± 12.8	0.33
Descending aorta length		210.2 ± 22.4	210.6 ± 23.9	0.93
Left carotid diameter		8.9 ± 1.7	8.3 ± 1.4	**0.049**
Brachiocephalic‐left carotid distance		2.9 ± 2.8	2.0 ± 2.4	0.05
Left carotid‐left subclavian distance		7.0 ± 3.6	9.1 ± 4.7	**0.01**
Incidental finding		19 (59.4)	13 (40.6)	**<0.001**

*Note*: Values are reported as mean ± Standard deviation (range) or frequency (%).

Bold values indicate significant association.

**Table 5:** Evaluation of morphometric parameters measured in the patients based on diabetes.

Variable		Diabetes		*P*‐Value
		Yes; *n* = 28	No; *n* = 133	
Age (years) mean Std. deviation		61.4 ± 16.4	56.1 ± 19.8	0.19
Sex	Male	18 (16.2)	93 (83.8)	0.83
	Female	10 (35.7)	43 (31.6)	
Type of Aortic Arch	One	8 (12.7)	55 (87.3)	**0.04**
	Two	18 (26.9)	49 (73.1)	
	Three	1 (5.6)	17 (94.4)	
Bovine Arch	Yes	6 (11.3)	47 (88.7)	0.19
	No	22 (21.2)	82 (78.8)	
Proximal part of ascending aorta		30.3 ± 5.6	32.5 ± 27.9	0.69
Proximal part of descending aorta		24.0 ± 3.1	24.7 ± 4.5	0.42
Distal part of ascending aorta		31.7 ± 5.4	31.4 ± 4.4	0.73
Distal part of descending aorta		21.5 ± 2.9	22.9 ± 4.0	0.10
Brachiocephalic diameter		13.3 ± 2.3	13.4 ± 2.2	0.93
Left subclavian diameter		11.4 ± 3.0	10.9 ± 2.3	0.29
Angle of the arch of aorta		64.9 ± 5.2	66.4 ± 7.3	0.33
Ascending aorta length		61.3 ± 13.3	63.7 ± 11.8	0.36
Descending aorta length		206.4 ± 19.7	211.4 ± 24.2	0.32
Left carotid diameter		8.8 ± 1.5	8.4 ± 1.5	0.19
Brachiocephalic‐left carotid distance		2.1 ± 2.0	2.3 ± 2.7	0.77
Left carotid‐left subclavian distance		7.2 ± 4.7	8.9 ± 4.4	0.08
Incidental finding		4 (14.3)	24 (18.6)	0.46

*Note*: Values are reported as mean ± Standard deviation (range) or frequency (%).

Bold values indicate significant association.

**Figure 1:**
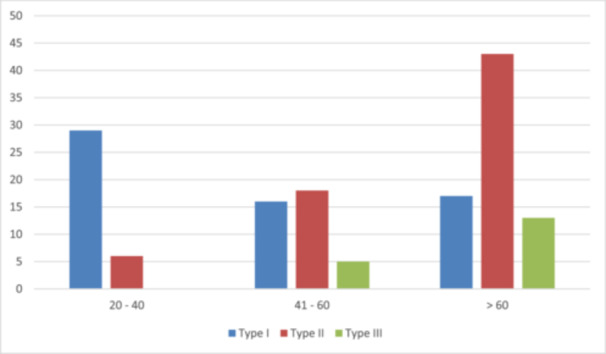
Brachiocephalic artery detachment based on age groups. Determining the relationship between brachiocephalic artery detachment based on three‐level division with the age of patients.

Figure 1 demonstrates the relationship between brachiocephalic artery detachment based on a three‐level division with the age of patients. There was a significant association in this regard (*p* < 0.001), in which the majority of patients with type I arch were among the 20‐ to 40‐year‐old age group (82.9%). A decrease in the frequency of type I arch and also a significant increase in the frequency of type 2 and 3 arches were observed with the increase of age (Pearson correlation = 0.489; *p* < 0.001), in which the majority of type III arch was observed in patients above 60 years old (72.2%).

**Figure 2:**
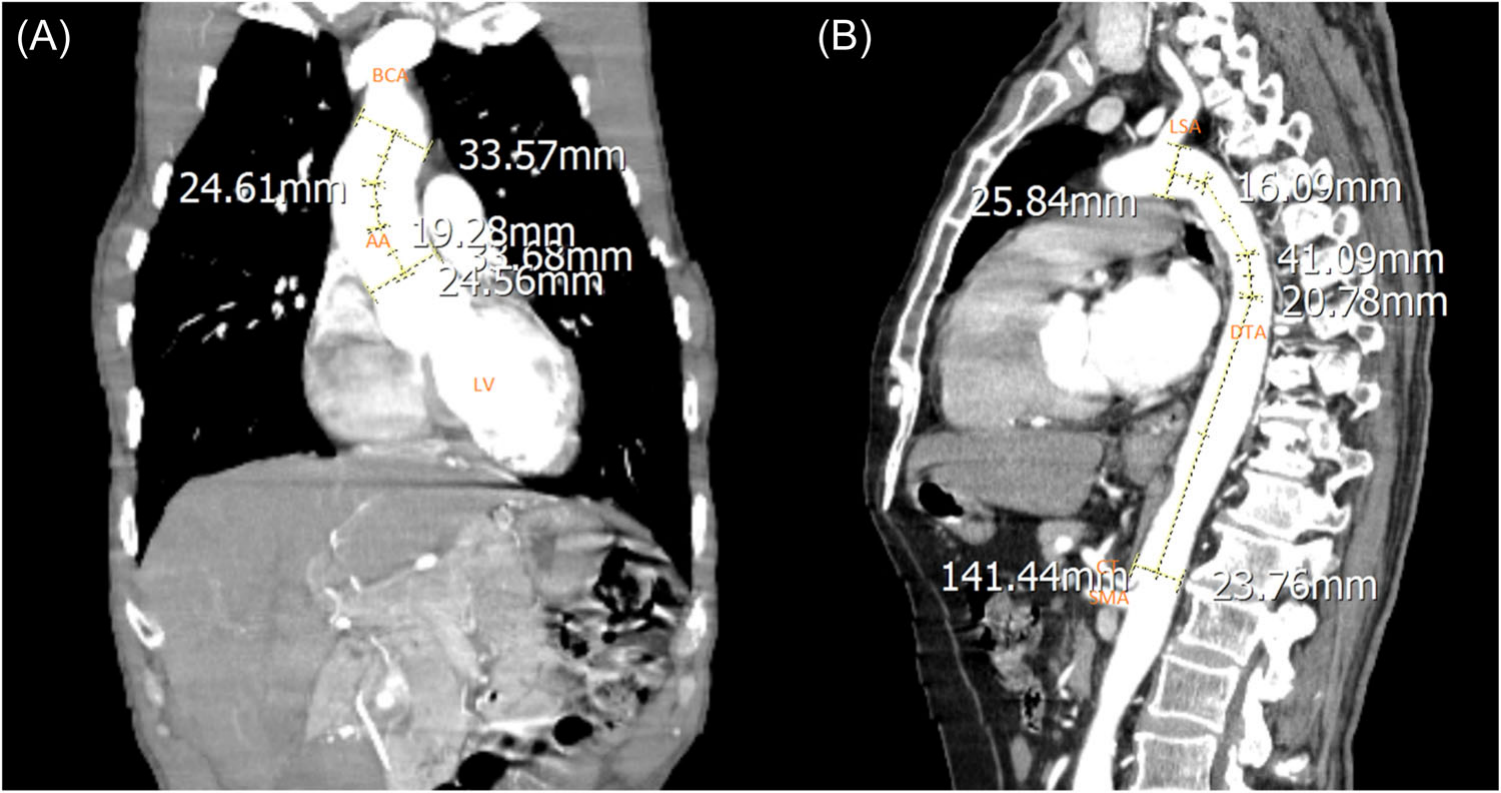
Computed tomography scan with Coronal (A) and Sagittal (B) view, demonstrating applied measurements in our study. (A) The length of the ascending aorta from the sinotubular junction to the brachiocephalic detachment and the diameter of the ascending aorta at the proximal and distal end; descending (B) The length of the descending aorta and the diameter of the descending aorta at the proximal and distal end (AA: Ascending aorta; DTA: descending thoracic aorta; BCA: brachiocephalic artery; LCC: left common carotid artery; LSA: left subclavian artery; LV: left ventricle; PA: pulmonary artery; LPA: left pulmonary artery; RPA: right pulmonary artery; CA: celiac artery; SMA: superior mesenteric artery).

**Figure 3:**
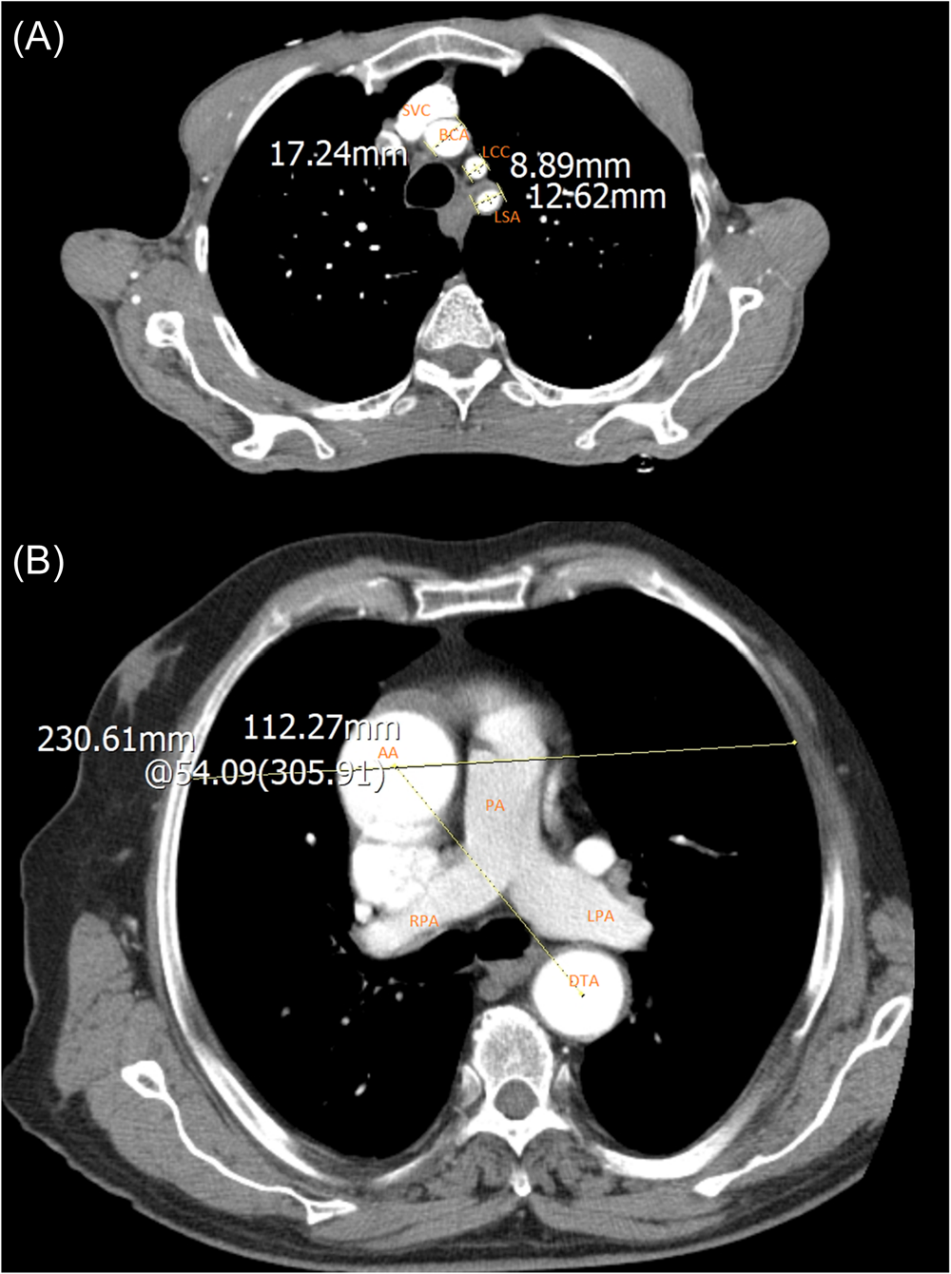
Computed tomography scan with Axial view, demonstrating applied measurements in our study. (A) The diameter of the brachiocephalic artery and the diameter of the left carotid artery at the proximal end; (B) The angle of curvature of the aortic arch relative to the coronal plane at the carina (demonstrating 62.38 degrees) (AA: Ascending aorta; DTA: descending thoracic aorta; BCA: brachiocephalic artery; LCC: left common carotid artery; LSA: left subclavian artery; LV: left ventricle; PA: pulmonary artery; LPA: left pulmonary artery; RPA: right pulmonary artery; CA: celiac artery; SMA: superior mesenteric artery).

**Figure 4:**
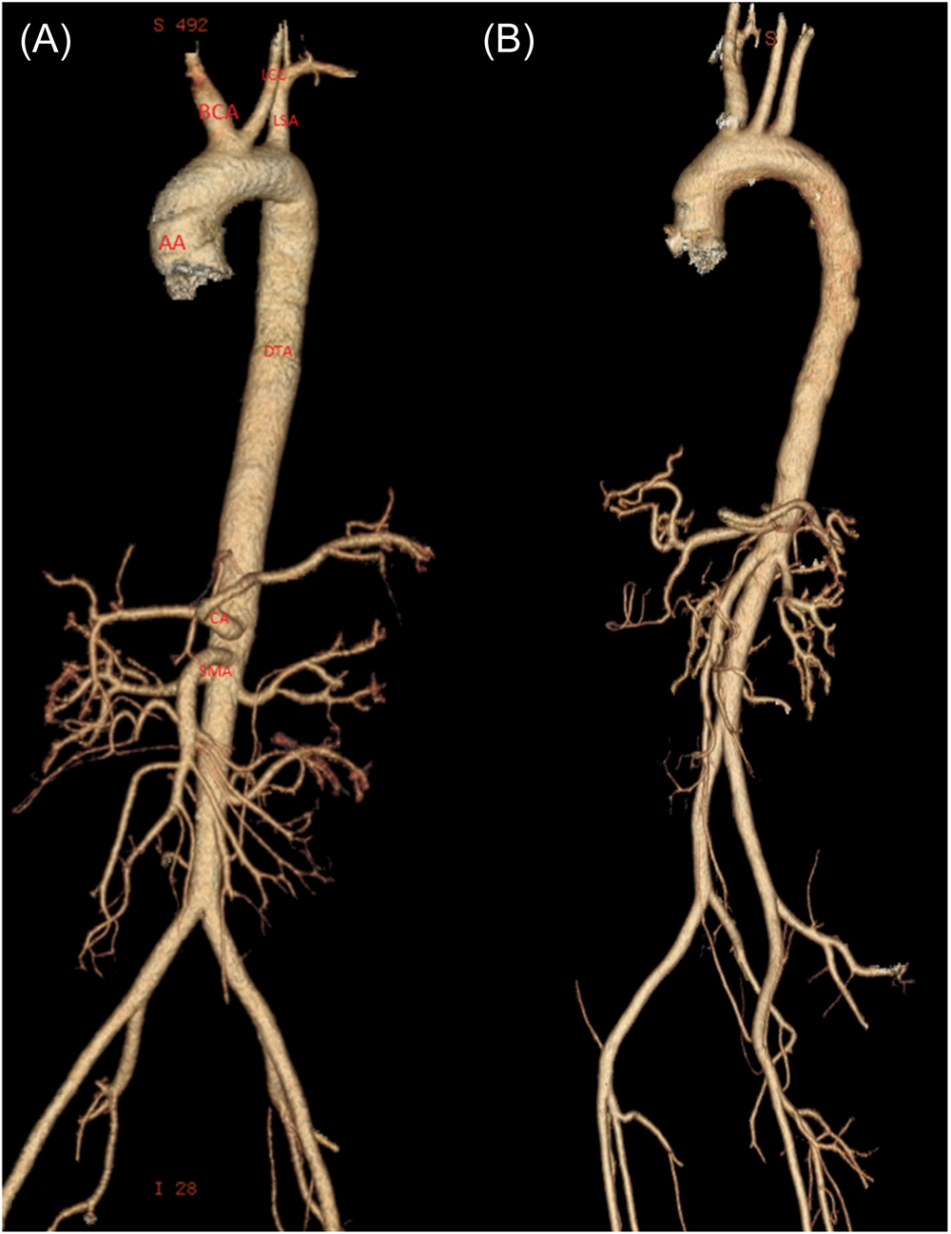
Computed tomography scan reconstruction of patient demonstrating Bovine arch. Computed tomography angiography demonstrating a patient with (A) and without (B) Bovine Arch. (AA: Ascending aorta; DTA: descending thoracic aorta; BCA: brachiocephalic artery; LCC: left common carotid artery; LSA: left subclavian artery; LV: left ventricle; PA: pulmonary artery; LPA: left pulmonary artery; RPA: right pulmonary artery; CA: celiac artery; SMA: superior mesenteric artery).

Figures 2, 3 and 4 illustrates the measurements among several cases in our study.

Regarding the incidental findings in our study, there were two cases in which the vertebral artery originated from the AA. Seven cases presented ascending aortic aneurysms; in one, dissection of the aorta was seen concurrently. Three cases had dissection of the aorta without aortic aneurysms. Two patients were diagnosed with descending aortic coarctation; one had an aberrant RSA. There were also three other cases of the aberrant RSA. We also observed two cases of left subclavian occlusion, and also two left common carotid occlusions.

## 4 DISCUSSION

The prime intention of the current study was to investigate the frequency of AA branching patterns and the association between morphologic features of AA and age using CTA of adult patients. These types of anatomical variations are generally asymptomatic and diagnosed incidentally. Morphologic features of the thoracic aorta, including the length and diameter, the origin of the vessels arising from the aorta, the distances between them, and AA angle, have great importance during performing aortic endovascular procedures or open vascular surgeries. Thus, diagnosis of AA branching variations before interventional procedures is required to optimize device deployment and prevent complications that may occur during the procedures; Otherwise, it may lead to irreversible consequences for persistent endo‐leak, ischemic complications of the brain and upper extremities.^11^, ^12^, ^13^, ^25^ To our best of our knowledge, despite the wide range of AA variability, the incidence of variation in this particular anatomical site has been surveyed by few studies, which mainly compromise post‐mortem examinations or include a limited number of cases.^25^, ^26^

CTA and MR‐Angiography are noninvasive methods displaying a detailed view of vascular diseases. For evaluating the thoracic aorta, CTA is considered an advantageous method compared to catheter angiography, as it enables the clinician to diagnose vascular anomalies; furthermore, this procedure is less invasive than catheter angiography and can be performed in outpatient settings as well. This method has increasingly been used to assess the morphology of the AA.^27^ Concerning the fact that successful endovascular AA surgeries and the descending aorta, such as TEVAR, are contingent on seal and fixation of the proximal graft, diverse morphologic curvatures of AA may pose an obstacle toward ideal deployment. An adaptive optimization algorithm based on CTA data is required before TEVAR to determine the clinically best C‐arm angle to use during the intervention.^28^

In the present population, the most common variant of arch anatomy was reported to be the “bovine” arch, with a prevalence of roughly 33.7% of our cases. This result concurs with other studies performed by Jakanani et al. and Recto et al., which established that 20% and 10.1% of their cases had a bovine arch, respectively.^20^, ^21^ An earlier study showed a greater proportion (∼40%) of the bovine pattern in African Americans.^29^ It appears that race contributes to the variations of arch branches. Additionally, there was a statically positive relationship between the mean maximal mean from the LSA to LCCA and patients with this particular pattern. While the mentioned distance was significantly higher among patients with bovine arch, the lumen diameter of the LCCA was significantly lower. Furthermore, no association was found between the bovine arch pattern and comorbid diseases, including hypertension or diabetes.

Mortality risks from TEVAR are greatly attributed to timing and age. Compared to younger patients, those over 75 years of age have three to five times the mortality risk following urgent or emergent TEVAR.^30^ Previous studies claimed that younger age and small aortic diameter are the two considerable key factors leading to successful TEVAR.^30^ According to our results, aging directly impacted aortic measurements. The inner diameter at the end of the aorta and the length of the ascending and descending aorta represented direct correlations with age. In other words, the aorta is enlarging in diameter and length during its lifetime.^31^, ^32^ Mismatching between the aortic diameter and the endograft could theoretically result in increased risks of endograft‐related complications such as endograft migration, endo‐leak, aneurysm growth, device failure, rupture, or re‐intervention.

In endovascular surgeries, the diameter of the AA branches within the landing zone segment is required for the insertion of a guiding catheter within a major branch, and the inner diameter of the blood vessel should be considered. The inner diameter of the major branches may vary and pose a significant risk of endo‐leak or failure of a proximal fixation; our study demonstrated that the lumen diameters of the BCT and LCCA were substantially higher in patients with hypertension compared to the normotensive cases while the mean maximal distance between the LSA and the LCCA was significantly lower in hypertensive cases. Laplace's law states that tangential stress is directly proportional to applied pressure and vessel radius and inversely proportional to wall thickness.^33^

We utilized the sagittal view for assessing this distance due to its optimal perspective. This particular distance is essential to be approximated since the application of the traditional TEVAR requires a proximal and distal landing zone of at least 2 cm.^24^

Aortic seal zone measurement in the angulated arch requires assessment of more than one criterion like the length. The first fundamental area for achieving seal and preventing bird‐beaking is the inner curvature apposition.^31^ Our survey revealed that preoperative AA curvature ‐which was the angle formed by AA to the coronal plane in axial view (left anterior oblique angle of C‐arm during angiography) at the level of carina‐ measurements had a remarkable difference between the minimal and maximal values of AA angle (43.00° and 87.00°) with average 66° ∓ 7.01° SD. In a study by Shin et al. average AA angle to the coronal plane was 62.2°.^34^ Additionally, the AA angle was significantly and positively correlated to aging (*p* < 0.05, coefficient correlation = 0.163). In patients with severe curvature at the AA, generous endograft oversizing may be required to ensure adequate apposition along the inner curvature to prevent a type I endo‐leak or graft migration. Meanwhile, too much oversizing may increase the risk of retrograde type A dissection and may cause stent‐graft infolding or accelerated aneurysm degeneration.^35^ A proximal seal zone in the AA is commonly essential during the stent graft deployment in the TEVAR procedure. Extreme angulation or curvature of the proximal landing zone segment can result in incomplete endograft apposition to the aortic lumen wall (bird‐beak configuration).^36^ Other types of AA angulations were evaluated in earlier studies,^31^, ^37^ which also showed a vast spectrum among two extremities. The optimal angiographic viewing angle estimated in the current study for observing AA is 66° Left Anterior Oblique (LAO). Notably, previous surveys have not dealt with AA angle at this exact sitem, therefore further comparison was not possible.

Regarding the incidental findings in the present study, six patients had undergone TEVAR. There were two cases in which vertebral artery originated directly from the AA. Seven of them presented ascending aortic aneurysms, and one showed dissections of the aorta concurrently. In contrast, three patients had dissection of the aorta without aortic aneurysms. Additionally, two were diagnosed with descending aortic coarctation, which one of them had aberrant RSA. Also, three other cases of aberrant RSA, two of LSA occlusion, and two LCCA occlusions were found.

### 4.1 Limitations

Even though the current survey is not a population‐based study, our center is the single referral center for vascular surgery in our region; therefore, it is safe to say that our study group can be generalized to the general population; however, further multicentral studies are justified. In the present study, there was no attempt to correlate the results with the ethnic origin of the patients, as it would have been difficult to collect these data retrospectively. However, the results of our study reflect the prevalence of each variation in diverse ethnic backgrounds since the imaging was done on a large geographic region.

## 5 CONCLUSION

Evaluating the AA and its orientations during TEVAR and conventional open surgeries is essential to case planning. We were able to find the geometrical features of AA and branches for inserting catheters into the AA and its major branches by studying 164 Iranian adults. Precisely, a clear understanding of AA pattern is strongly determined as the preferred method with lower morbidity and mortality.

## AUTHOR CONTRIBUTIONS

**Ahmad Hosseinzadeh**: Conceptualization; Supervision. **Ali Tajaddini**: Conceptualization; Investigation. **Seyed Hamed Jafari**: Investigation; Data curation. **Zahra Mohammadi**: Writing—original draft. **Farzad Dalfardi**: Validation. **Hossein Fatemian**: Data curation. **Reza Shahriarirad**: Investigation; Formal analysis; Project administration; Writing—review and editing; Data curation; Software.

## CONFLICT OF INTEREST STATEMENT

The authors declare no conflicts of interest.

## ETHICS STATEMENT

The study was approved by the Research Ethics Committee of the Shiraz University of Medical Sciences and all data have been gathered in accordance with the ethical standards. Patients' information was anonymized before analysis and their confidentiality were kept by the researcher. Furthermore, all methods were carried out in accordance with relevant guidelines and regulations and all ethical principles of the Declaration of Helsinki were considered.

## TRANSPARENCY STATEMENT

The lead author Reza Shahriarirad affirms that this manuscript is an honest, accurate, and transparent account of the study being reported; that no important aspects of the study have been omitted; and that any discrepancies from the study as planned (and, if relevant, registered) have been explained.

## Data Availability

The authors confirm that the data supporting the findings of this study are available within the article [and/or] its supplementary materials. The datasets used and/or analyzed during the current study are also available from the corresponding author on reasonable request.
